# Complete Genome Sequences of Streptococcus mitis Strains Isolated from the Oral Cavity and Urogenital Tract of a Woman and Her Male Sexual Partner

**DOI:** 10.1128/MRA.01379-19

**Published:** 2020-01-02

**Authors:** Carine R. Mores, Travis K. Price, Bridget Brassil, Catherine Putonti, Alan J. Wolfe

**Affiliations:** a Department of Microbiology and Immunology, Stritch School of Medicine, Loyola University Chicago, Maywood, Illinois, USA; b Department of Biology, Loyola University Chicago, Chicago, Illinois, USA; c Bioinformatics Program, Loyola University Chicago, Chicago, Illinois, USA; d Department of Computer Science, Loyola University Chicago, Chicago, Illinois, USA; University of Maryland School of Medicine

## Abstract

Streptococcus mitis is a member of the mitis group of the genus Streptococcus, which includes commensal species of the oral cavity and upper respiratory tract. Here, we report 39 complete genome sequences of S. mitis strains isolated from the oral cavity and urogenital tract of a woman and her male sexual partner.

## ANNOUNCEMENT

Streptococcus mitis is an abundant member of the commensal microbiota of the upper respiratory tract and oral cavity (1, 2). Although it is considered to be a commensal species, S. mitis can cause a variety of invasive diseases in human (3). In order to increase our knowledge of this commensal bacterium, we isolated multiple strains of S. mitis from a woman and her male sexual partner. These strains were isolated from different anatomical sites and on different days. Here, we present the genome sequences for a subset of this collection.

Samples were collected from oral swabs, vaginal swabs, periurethral swabs, penile swabs, and voided urine samples from one female and her male sexual partner as part of an institutional review board (IRB)-approved study (LU 209830). Swabs were collected using the BD liquid Amies elution swab (ESwab) collection system. Urine samples were collected as clean-catch midstream voided urine. (Note that the participant was given instructions for obtaining the voided urine samples, and the periurethral swab and urine samples differed significantly [4].) Strains were isolated using a modified version of the expanded quantitative urine culture (EQUC) protocol (5) and stored at −80°C. From these samples, 39 S. mitis strains, identified by matrix-assisted laser desorption ionization–time of flight (MALDI-TOF) mass spectrometry, were selected for whole-genome sequencing. Each S. mitis isolate was grown on Columbia colistin-naladixic acid agar with 5% sheep blood plates incubated under 5% CO_2_ conditions at 37°C for 48 h. Pure cultures of S. mitis were transferred to brain heart infusion (BHI) broth with 10% fetal bovine serum (FBS) and incubated under 5% CO_2_ conditions at 37°C for 48 h and pelleted. DNA was extracted from pellets using a phenol-chloroform method and quantified using a Qubit 2.0 fluorometer and an Agilent Bioanalyzer. DNA libraries were created using the Illumina Nextera kit and sequenced using the MiSeq reagent kit v2, producing, on average, 336,117 pairs of 250-bp reads. Quality control and demultiplexing of sequence data were done with onboard MiSeq Control software and MiSeq Reporter v3.1. Raw reads were trimmed using Sickle v1.33 (https://github.com/najoshi/sickle) and assembled using SPAdes v3.13.0 (6) with the “only-assembler” option for k values of 55, 77, 99, and 127. Genome coverage was calculated using BBMap v38.47 (https://sourceforge.net/projects/bbmap/). The NCBI Prokaryotic Genome Annotation Pipeline (PGAP) v4.8 (7) was used to annotate the genome sequences. Unless previously noted, default parameters were used for each software tool.

Table 1 lists all of the 39 S. mitis strains and their source, as well as their genome assembly statistics. The average GC content is 41%, similar to that reported in GenBank for other strains of the species. Annotations identified an average of 1,985 coding sequences (CDS) (Table 1). The strains varied in their numbers of rRNA operons and tRNAs. The addition here of 39 genome sequences greatly increases our knowledge of the genetic diversity of this bacterial species within the oral and lower urinary tract microbiota.

**TABLE 1:** Genome assembly and annotation statistics

Strain	Partner sex	Isolation source	Coverage (×)	No. of contigs	*N*_50_ (bp)	Genome length (bp)	No. of CDS	No. of rRNAs	No. of tRNAs	GenBank WGS accession no.^*a*^	SRA accession no.
SM05	Female	Voided urine sample	59.22	23	194,401	2,001,354	1,950	3	44	WIKE00000000	SRR10341581
SM07	Female	Periurethral swab	64.74	27	199,509	2,002,948	1,956	3	44	WIKD00000000	SRR10341580
SM09	Female	Oral swab	68.4	15	187,601	1,973,655	1,962	3	43	WIKC00000000	SRR10341605
SM12	Male	Oral swab	33.68	46	105,495	2,095,125	2,043	3	42	WIKB00000000	SRR10341594
SM20	Female	Vaginal swab	50.33	55	82,209	2,008,422	1,969	3	43	WIKA00000000	SRR10341584
SM26	Female	Oral swab	58.51	27	251,708	2,060,956	1,982	3	41	WIJZ00000000	SRR10341583
SM27	Female	Voided urine sample	53.29	27	174,586	2,001,505	1,957	3	44	WIJY00000000	SRR10341582
SM30	Female	Periurethral swab	51.27	28	199,508	2,007,955	1,965	3	44	WIJX00000000	SRR10341577
SM33	Male	Oral swab	15.19	55	74,863	2,093,429	2,047	3	39	WIJW00000000	SRR10341576
SM39	Female	Oral swab	46.12	127	27,218	2,015,490	2,042	3	47	WIJV00000000	SRR10341575
SM45	Female	Voided urine sample	44.13	25	148,327	2,002,001	1,953	3	44	WIJU00000000	SRR10341579
SM48	Male	Oral swab	19.61	45	105,607	2,094,194	2,038	3	42	WIJT00000000	SRR10341578
SM04	Female	Voided urine sample	51.52	21	225,775	2,000,773	1,956	3	44	WIJS00000000	SRR10341613
SM06	Female	Voided urine sample	59.03	27	188,320	2,002,707	1,957	3	44	WIJR00000000	SRR10341612
SM08	Female	Periurethral swab	51.75	18	205,523	1,996,012	1,960	3	44	WIJQ00000000	SRR10341611
SM10	Female	Oral swab	51.35	49	132,942	2,168,718	2,121	4	39	WIJP00000000	SRR10341610
SM11	Female	Oral swab	56.8	113	30,148	2,014,391	2,031	4	47	WIJO00000000	SRR10341609
SM13	Male	Oral swab	48.15	120	97,578	2,152,221	2,150	3	43	WIJN00000000	SRR10341608
SM14	Male	Oral swab	47.22	58	64,862	2,094,407	2,055	3	42	WIJM00000000	SRR10341607
SM15	Male	Penile swab	44.11	21	155,584	2,016,502	2,018	3	52	WIJL00000000	SRR10341606
SM16	Male	Penile swab	53.16	67	72,040	2,017,910	1,886	2	40	WIJK00000000	SRR10341604
SM28	Female	Voided urine sample	59.9	30	186,870	2,007,541	1,963	3	44	WIJJ00000000	SRR10341603
SM29	Female	Voided urine sample	47.58	24	199,508	2,005,875	1,961	3	44	WIJI00000000	SRR10341602
SM34	Male	Oral swab	18.72	46	105,662	2,091,923	2,047	3	43	WIJH00000000	SRR10341601
SM40	Female	Oral swab	53.86	124	27,218	2,015,048	2,039	3	47	WIJG00000000	SRR10341600
SM41	Female	Oral swab	46.7	27	177,356	2,032,459	2,058	3	44	WIJF00000000	SRR10341599
SM46	Female	Voided urine sample	52.34	28	187,795	2,027,642	1,964	3	44	WIJE00000000	SRR10341598
SM47	Female	Voided urine sample	46.19	31	147,586	2,006,587	1,965	3	44	WIJD00000000	SRR10341597
SM49	Male	Oral swab	64.52	30	141,856	1,996,076	1,958	2	44	WIJC00000000	SRR10341596
SM50	Male	Oral swab	49.28	18	208,384	1,894,166	1,859	4	49	WIJB00000000	SRR10341595
SM17	Female	Voided urine sample	33.99	450	7,154	1,984,878	1,944	2	37	WIJA00000000	SRR10341593
SM18	Female	Voided urine sample	137.48	30	147,586	2,000,922	1,954	3	44	WIIZ00000000	SRR10341592
SM19	Female	Voided urine sample	111.07	27	186,870	2,006,191	1,965	3	44	WIIY00000000	SRR10341591
SM35	Female	Voided urine sample	100.62	25	187,601	2,007,565	1,963	3	44	WIIX00000000	SRR10341590
SM36	Female	Voided urine sample	106	25	186,870	2,005,820	1,964	3	44	WIIW00000000	SRR10341589
SM37	Female	Voided urine sample	76.02	29	188,320	2,006,041	1,963	3	44	WIIV00000000	SRR10341588
SM42	Female	Voided urine sample	115.16	33	131,530	2,007,726	1,962	3	44	WIIU00000000	SRR10341587
SM43	Female	Voided urine sample	94.02	26	147,586	2,000,668	1,954	3	44	WIIT00000000	SRR10341586
SM44	Female	Voided urine sample	115.16	23	186,871	2,000,125	1,953	3	44	WIIS00000000	SRR10341585

a WGS, whole-genome shotgun.

### Data availability.

This whole-genome shotgun (WGS) project has been deposited in GenBank, and the accession numbers for each genome assembly are listed in Table 1. The versions described in this paper are the first versions. Raw sequence data are publicly available in SRA for the 39 S. mitis strains; the accession numbers are listed in Table 1. The WGS and SRA records are associated with BioProject number PRJNA316969.
